# Supplementary material for: Rhodopsin-positive cell production by intravitreal injection of small molecule compounds in mouse models of retinal degeneration
Source: PLoS One. 2023 Feb 23;18(2):e0282174. doi: 10.1371/journal.pone.0282174 (PMC9949636; doi:10.1371/journal.pone.0282174)
Supplement: S1 Table — (PDF) [file pone.0282174.s011.pdf]

S1 Table. Primer sequences

Gene	Forward	Reverse
(Mouse)		
B-actin	GATGACCCAGATCATG	GGAGAGCATAGCCCTC
Rho	TTGCCACACTTGGAGGTGAA	CCACCACGTAGCGCTCAAT
CD44	GGGACGGTGGAAGAGAGGAA	CCCAATCTTCATGTCCACAC
Ki67	AGGGTAACTCGTGGAACCAA	TTAACTTCTTGGTGCATACAATGTC
Crx	GTTCAAGAATCGTAGGGCGAA	TGAGATGCCCAAAGGATCTGT
RBPMS	GTACCCAGCGGAGTTAGCG	AAGACAGGTGTGTTGGGCTTT
Prox1	AGAAGGGTTGACATTGGAGTGA	TGCGTGTTGCACCACAGAATA
Islet1	ATGATGGTGGTTTACAGGCTAAC	TCGATGCTACTTCACTGCCAG
Short-wavelength Opsin	TGTACATGGTCAACAATCGGA	ACACCATCTCCAGAATGCAAG
Calbp2	AGTACACCCAGACCATACTACG	GGCCAAGGACATGACACTCTT
Tcf4	GGCTTGACCGACAGACTTTATG	GGGAGCCTAACAGATGCGG
PCP2	AGAAAGCCGTAAGCAGGGC	CCCCATCCTGAGTCCCAAGAT
Meis2	CAGGGTGGTCCAATGGGAATG	GGGGGTCCATGTCTTAACTGAG
Snap25	CAACTGGAACGCATTGAGGAA	GGCCACTACTCCATCCTGATTAT
ASCL1	GCAACCGGGTCAAGTTGGT	GTCGTTGGAGTAGTTGGGGG
HMGA1	CTCCAGGGAGGAAACCAAG	CAGAGGACTCCTGGGAGATG
SOX2	CATCCACTTCTACCCCACCTT	AGCTCCCTGTCAGGTCCTT
LTBP2	AACAGCACCAACCACTGTATC	CCTGGCATTCTGAGGGTCAAA
Id	CCTAGCTGTTCGCTGAAGGC	CTCCGACAGACCAAGTACCAC
Axin2	TGACTCTCCTTCCAGATCCCA	TGCCCACACTAGGCTGACA
DLL1	CAGGACCTTCTTTCGCGTATG	AAGGGGAATCGGATGGGGTT
Nxnl1	GAAGTGGAGACAGAGGCAGA	AAGAAGTCTTTGAGGACTGGG
(Rat)		
B-actin	CCCGCGAGTACAACCT	CGTCATCCATGGCGAACT
Rho	AACCTTGAGGGCTTCTTTGCCA	AAGTTGCTCATGGGCTTGCAGA
GS	TACCCGAGTGGAACTTTGATG	TAAAGTTGGTGTGGCAGCCTG
