# Supplementary material for: Rhodopsin-positive cell production by intravitreal injection of small molecule compounds in mouse models of retinal degeneration
Source: PLoS One. 2023 Feb 23;18(2):e0282174. doi: 10.1371/journal.pone.0282174 (PMC9949636; doi:10.1371/journal.pone.0282174)
Supplement: S2 Table — (PDF) [file pone.0282174.s012.pdf]

S2 Table. Lists of markers for the identification of retinal cells in qPCR

Markers	Retinal cell type	Reference
RBPMS	Ganglion cell	[22]
Prox1, Tcf4, Meis2	Amacrine cell	[23-24]
Islet1, PCP2	Bipolar cell	[25-26]
Calbp2, Snap25	Horizontal cell	[27-28]
