# Supplementary figures and images for: Rhodopsin-positive cell production by intravitreal injection of small molecule compounds in mouse models of retinal degeneration
Source: PLoS One. 2023 Feb 23;18(2):e0282174. doi: 10.1371/journal.pone.0282174 (PMC9949636; doi:10.1371/journal.pone.0282174)

The original image of Fig 1G

The image in the yellow frame is shown in Fig 1G.

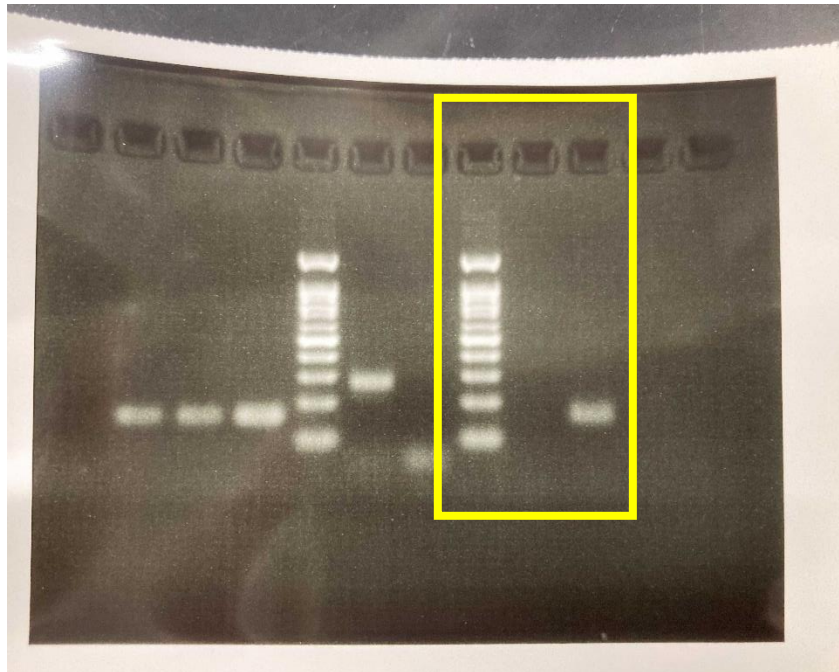

Supplement: S1 Data — (PDF) [file pone.0282174.s013.pdf]
