# Supplementary material for: Rhodopsin-positive cell production by intravitreal injection of small molecule compounds in mouse models of retinal degeneration
Source: PLoS One. 2023 Feb 23;18(2):e0282174. doi: 10.1371/journal.pone.0282174 (PMC9949636; doi:10.1371/journal.pone.0282174)
Supplement: S2 Data — (PDF) [file pone.0282174.s014.pdf]

Fig2

D	treatment	Rho - expressing retinal region (%)
	DMSO	0
	DMSO	8.359
	DMSO	1
	DMSO	8
	DMSO	4
	SLCD	27.89
	SLCD	32.67
	SLCD	23
	SLCD	10
	SLCD	12

E	treatment	Rho	Opsin
	DMSO	0.825989	0.920851
	DMSO	0.93187	0.726754
	DMSO	1.039373	0.374368
	DMSO	1.372916	2.342903
	DMSO	0.811185	0.634274
	SLCD	1.794629	2.492273
	SLCD	1.513493	3.19429
	SLCD	1.210323	1.257349
	SLCD	1.573498	0.959484
	SLCD	1.704676	1.021994

G	treatment	TUNEL - positive cells
	DMSO	89
	DMSO	107
	DMSO	70
	DMSO	85
	DMSO	90
	SLCD	100
	SLCD	110
	SLCD	90
	SLCD	99
	SLCD	104

J	treatment	Rho	Opsin	Prox1	Islet
	DMSO	1.192301	0.635893	0.727798	0.936448

DMSO	0.793523	1.257308	1.601108	0.407256
DMSO	1.014176	1.106798	0.671093	1.656295
DMSO	0.910765	1.188298	0.704459	1.756285
DMSO	1.176115	0.286454	0.714378	0.413416
SLCD	2.137667	1.60963	1.087309	0.984662
SLCD	1.821196	0.884452	0.001861	0.767602
SLCD	1.723039	1.146473	0.675352	0.885078
SLCD	1.88421	0.893393	1.558676	1.732457
SLCD	2.036588	0.541092	2.0584	0.258173
