# Supplementary material for: Rhodopsin-positive cell production by intravitreal injection of small molecule compounds in mouse models of retinal degeneration
Source: PLoS One. 2023 Feb 23;18(2):e0282174. doi: 10.1371/journal.pone.0282174 (PMC9949636; doi:10.1371/journal.pone.0282174)
Supplement: S3 Data — (PDF) [file pone.0282174.s015.pdf]

Fig5

B	treatment	Rho	Opsin
	DMSO	0.710249	0.399589
	DMSO	0.835164	1.608846
	DMSO	1.015791	1.004218
	DMSO	1.222668	1.461955
	DMSO	1.216125	0.525392
	SLCD	1.675071	0.335042
	SLCD	1.443015	0.524852
	SLCD	1.36396	0.507557
	SLCD	2.084698	0.872998
	SLCD	1.75761	1.004218

E	treatment	Rho - expressing retinal region (%)
	DMSO	2
	DMSO	5
	DMSO	6
	DMSO	8
	DMSO	4.6
	SLCD	28
	SLCD	30.5
	SLCD	20
	SLCD	22
	SLCD	24.1

G	treatment	PNA - positive cells
	DMSO	134.25
	DMSO	148.9167
	DMSO	138.5
	DMSO	150
	DMSO	135.25
	SLCD	171.6667
	SLCD	193.75
	SLCD	184.8333
	SLCD	160
	SLCD	180

H	treatment	Nxnl1
	DMSO	0.507127

DMSO	0.639489
DMSO	1.259393
DMSO	1.192349
DMSO	1.401663
SLCD	2.635513
SLCD	2.128843
SLCD	2.520089
SLCD	2.483476
SLCD	2.011851
