# Supplementary material for: Rhodopsin-positive cell production by intravitreal injection of small molecule compounds in mouse models of retinal degeneration
Source: PLoS One. 2023 Feb 23;18(2):e0282174. doi: 10.1371/journal.pone.0282174 (PMC9949636; doi:10.1371/journal.pone.0282174)
Supplement: S4 Data — (PDF) [file pone.0282174.s016.pdf]

Fig6

B	treatment	a - wave (μ v)
	DMSO	334
	DMSO	303
	DMSO	291
	DMSO	302
	DMSO	142
	DMSO	286
	DMSO	144
	DMSO	234
	SLCD	344
	SLCD	299
	SLCD	315
	SLCD	375
	SLCD	335
	SLCD	375
	SLCD	360
	SLCD	325
	SLCD	334
	SLCD	303
