# Supplementary material for: Rhodopsin-positive cell production by intravitreal injection of small molecule compounds in mouse models of retinal degeneration
Source: PLoS One. 2023 Feb 23;18(2):e0282174. doi: 10.1371/journal.pone.0282174 (PMC9949636; doi:10.1371/journal.pone.0282174)
Supplement: S5 Data — (PDF) [file pone.0282174.s017.pdf]

S2 Fig

treatment	LTBP1	Id	Axin2	DLL1
DMSO	0.253622	0.801761	1.129131	0.681716
DMSO	0.201319	1.428565	0.972936	0.755956
DMSO	2.54506	0.553964	0.897947	0.918238
DMSO	1.36744	0.874792	1.651366	1.669433
DMSO	2.211176	0.952376	1.169368	0
SLCD	0	0.154999	8.693074	0
SLCD	0	0.244667	9.383491	0
SLCD	0	0.064907	8.002656	0
SLCD	0	0.077641	7.075381	0
SLCD	0	0.169047	8.512224	0
