# Supplementary material for: Rhodopsin-positive cell production by intravitreal injection of small molecule compounds in mouse models of retinal degeneration
Source: PLoS One. 2023 Feb 23;18(2):e0282174. doi: 10.1371/journal.pone.0282174 (PMC9949636; doi:10.1371/journal.pone.0282174)
Supplement: S6 Data — (PDF) [file pone.0282174.s018.pdf]

S3 Fig

treatment	Ascl1	HMGA1	SOX2
DMSO (Da	1.203678	1.506212	1.077134
DMSO (Da	0	0.915832	1.012049
DMSO (Da	0.919921	0.577959	0.910817
DMSO (Da	2.180834	1.078559	0.997713
DMSO (Da	0.695593	0.963115	0.828236
SLCD (Day	3.533252	2.270081	1.947764
SLCD (Day	2.30184	1.660095	1.449549
SLCD (Day	2.184756	2.389427	1.441914
SLCD (Day	3.247473	2.236333	2.529014
SLCD (Day	2.293397	2.196342	2.051097
DMSO (Da	1.558265	1.016107	0.870271
DMSO (Da	0.817511	0.973935	0.910079
DMSO (Da	0.624225	0.944955	0.727822
DMSO (Da	0.800887	1.066842	1.045808
DMSO (Da	0.62846	0.947091	0.637872
SLCD (Day	4.918283	1.503051	0.787762
SLCD (Day	1.662096	1.618419	0.844837
SLCD (Day	6.009276	1.618448	0.817295
SLCD (Day	12.90048	1.404742	0.916425
SLCD (Day	7.648793	1.401574	1.999416
