# Supplementary material for: Rhodopsin-positive cell production by intravitreal injection of small molecule compounds in mouse models of retinal degeneration
Source: PLoS One. 2023 Feb 23;18(2):e0282174. doi: 10.1371/journal.pone.0282174 (PMC9949636; doi:10.1371/journal.pone.0282174)
Supplement: S7 Data — (PDF) [file pone.0282174.s019.pdf]

S4 Fig

treatment	RBPMS	Prox1	Islet1	Snap	PCP2	Tcf4	Meis2
DMSO	1.761208	0.831854	0.150151	1.747831	0.627321	0.449672	1.026754
DMSO	1.123064	1.113576	0.319766	1.502212	0.63786	0.449089	1.0597
DMSO	1.130188	1.315439	0.095235	1.700094	0.570977	0.453216	1.122527
DMSO	0.583646	0.740905	1.999409	0.014489	1.77005	1.701404	0.827569
DMSO	0.401894	0.998225	2.435438	0.031424	1.393793	1.946624	0.96345
SLCD	4.471976	0	0	0.159111	0.627321	0.517174	0.394092
SLCD	0	3.09228	1.042369	0.184002	0.63786	0.578706	1.06057
SLCD	4.191226	4.726043	1.956484	0.258369	0.570977	0.592095	0.611494
SLCD	0	1.202307	0.842167	0.384409	1.77005	0.435764	0.786161
SLCD	2.431884	5.054348	0.67933	0.293795	1.393793	0.337495	0.399778
