# Supplementary material for: Rhodopsin-positive cell production by intravitreal injection of small molecule compounds in mouse models of retinal degeneration
Source: PLoS One. 2023 Feb 23;18(2):e0282174. doi: 10.1371/journal.pone.0282174 (PMC9949636; doi:10.1371/journal.pone.0282174)
Supplement: S8 Data — (PDF) [file pone.0282174.s020.pdf]

S6 Fig

treatment	RBPMS	Prox1	Islet1	Opsin	Calbp2
DMSO (Da	1.198915	0.927061	1.081499	1.818564	1.03365
DMSO (Da	0.78525	1.020459	0.980929	1.073038	0.939805
DMSO (Da	0.940504	1.028467	0.789518	0.975678	0.993755
DMSO (Da	1.075333	1.024014	1.148056	1.13979	1.032791
DMSO (Da	1.10633	1.020459	1	1.251768	1.030058
SLCD (Day	1.208573	1.430984	1.253728	1.228525	1.323111
SLCD (Day	1.266769	0.966302	0.722647	1.006151	1.188752
SLCD (Day	0.787423	0.922145	0.78004	1.773892	0.734328
SLCD (Day	0.869107	1.018992	0.782363	1.233463	0.930247
SLCD (Day	1.015404	1	1.182858	1.173936	1.367008
DMSO (Da	0.937726	1.049314	1.176229	1.464504	1.425095
DMSO (Da	0.960685	1.028903	1.291457	1.222298	1.155473
DMSO (Da	0.663279	1.083346	1.389845	0.872408	1.969824
DMSO (Da	1.024592	1.232253	1.605958	0.443323	1.254862
DMSO (Da	1.118832	1.083346	1.440092	1.040193	1.784939
SLCD (Day	0.974708	1.243138	1.88676	2.098104	1.496136
SLCD (Day	0.949895	1.062812	1.600852	1.216971	1.370923
SLCD (Day	1.000808	1.017527	1.426349	1.865026	1.384599
SLCD (Day	1.440694	1.179539	1.57514	2.506918	1.467055
SLCD (Day	0.993671	1.062812	1.300319	1.552542	1.597468
