# Supplementary material for: Rhodopsin-positive cell production by intravitreal injection of small molecule compounds in mouse models of retinal degeneration
Source: PLoS One. 2023 Feb 23;18(2):e0282174. doi: 10.1371/journal.pone.0282174 (PMC9949636; doi:10.1371/journal.pone.0282174)
Supplement: S9 Data — (PDF) [file pone.0282174.s021.pdf]

S8 Fig

treatment	RBPMS	Prox1	Islet1	Opsin	Calbp2
DMSO (Da	0.812525	0.970516	1.112015	0.47745	0.667014
DMSO (Da	1.187475	1.251497	1.138648	1.922332	1.332986
DMSO (Da	0.812525	1.000214	0.682322	1.199891	0.667014
DMSO (Da	1.187475	0.777772	1.067015	1.746819	1.332986
DMSO (Da	0.836842	1.064347	0.656885	1.171867	1.313483
SLCD (Day	0.622876	0.674332	0.860256	0.400325	0.344734
SLCD (Day	1.527411	0.783815	1.143999	0.627121	0.485235
SLCD (Day	0.825393	0.710663	0.978752	0.606455	0.636428
SLCD (Day	1.003399	0.878029	1.164669	1.043103	0.633353
SLCD (Day	0.860489	0.77619	0.861657	1.092179	0.633393
DMSO (Da	0.819125	0.846874	1.196036	0.128819	0.616395
DMSO (Da	0.79898	0.885788	1.030656	0.179737	0.590902
DMSO (Da	1.072051	0.786179	1.354764	0.312294	0.901007
DMSO (Da	0.675341	0.840172	1.176337	0.496565	0.855347
DMSO (Da	1.002633	0.913172	1.057058	0.287406	0.875908
SLCD (Day	0.985169	1.003559	1.088521	0.143901	0.695363
SLCD (Day	0.878318	0.737231	0.89993	0.111262	0.641346
SLCD (Day	0.98579	0.843621	1.259278	0.327772	0.87625
SLCD (Day	0.824008	0.930997	1.193955	0.323523	0.894974
SLCD (Day	0.997481	0.923894	0.914549	0.201327	0.8132
