# Supplementary material for: Rhodopsin-positive cell production by intravitreal injection of small molecule compounds in mouse models of retinal degeneration
Source: PLoS One. 2023 Feb 23;18(2):e0282174. doi: 10.1371/journal.pone.0282174 (PMC9949636; doi:10.1371/journal.pone.0282174)
Supplement: S10 Data — (PDF) [file pone.0282174.s022.pdf]

Fig1

D	treatment	Rho - positive cells (%)
	DMSO	2
	DMSO	8
	DMSO	4.6
	DMSO	5
	DMSO	6
	SLCD	28
	SLCD	30.5
	SLCD	20
	SLCD	12
	SLCD	15

F	treatment	Rho	CD44
	DMSO	0.413355286	1.183005
	DMSO	0.951551312	0.788428
	DMSO	1.635093012	1.176401
	DMSO	0.790076121	1.086334
	DMSO	0.988471846	0.765831
	SLCD	37.12772049	0.133425
	SLCD	30.90306574	0.013127
	SLCD	20.18528412	0.0357
	SLCD	15.03051458	0.060395
	SLCD	9.739134379	0.107479

H	treatment	Rho	GS
	DMSO (Day1)	1.139236797	0.578881
	DMSO (Day1)	0.986891081	0.59161
	DMSO (Day1)	0.610322781	0.462392
	DMSO (Day1)	1.147749912	1.328821
	DMSO (Day1)	1.115799706	2.038301
	SLCD (Day1)	3.218740436	4.62026
	SLCD (Day1)	2.657221297	4.416026
	SLCD (Day1)	2.739101801	3.349011
	SLCD (Day1)	1.814062845	1.57886
	SLCD (Day1)	2.02047788	0.93727
	DMSO (Day3)	0.69882762	1.144979
	DMSO (Day3)	0.844832666	1.471618
	DMSO (Day3)	0.591647373	0.5969

DMSO (Day3)	0.936699254	0.731281
DMSO (Day3)	0.879047057	1.055213
SLCD (Day3)	4.242475954	0
SLCD (Day3)	1.071163636	0.13606
SLCD (Day3)	2.141880412	0.207648
SLCD (Day3)	2.260207094	0.119054
SLCD (Day3)	1.10184995	0.179442
