# Supplementary material for: Rhodopsin-positive cell production by intravitreal injection of small molecule compounds in mouse models of retinal degeneration
Source: PLoS One. 2023 Feb 23;18(2):e0282174. doi: 10.1371/journal.pone.0282174 (PMC9949636; doi:10.1371/journal.pone.0282174)
Supplement: S11 Data — (PDF) [file pone.0282174.s023.pdf]

Fig4

B

treatment	a - wave (μv)
DMSO	291
DMSO	302
DMSO	142
DMSO	286
DMSO	357
DMSO	320
DMSO	144
DMSO	234
SLCD	391
SLCD	344
SLCD	299
SLCD	315
SLCD	375
SLCD	335
SLCD	420
SLCD	375
SLCD	360
SLCD	325
SLCD	334
SLCD	303
