# Supplementary material for: Rhodopsin-positive cell production by intravitreal injection of small molecule compounds in mouse models of retinal degeneration
Source: PLoS One. 2023 Feb 23;18(2):e0282174. doi: 10.1371/journal.pone.0282174 (PMC9949636; doi:10.1371/journal.pone.0282174)
Supplement: Attachment — Submitted filename: Response_to_Reviewers.docx [file pone.0282174.s025.docx]

**A point-by-point response to the issues raised by the Editorial office and Reviewers**

We would like to thank the editorial office and reviewers for their insightful comments that have helped us improve the manuscript. We have addressed the reviewers’ comments in a point-by-point fashion and have summarized the specific changes as highlighted text in the revised manuscript.

**Journal Requirements:**

1. Please review your reference list to ensure that it is complete and correct. If you have cited papers that have been retracted, please include the rationale for doing so in the manuscript text, or remove these references and replace them with relevant current references. Any changes to the reference list should be mentioned in the rebuttal letter that accompanies your revised manuscript. If you need to cite a retracted article, indicate the article’s retracted status in the References list and also include a citation and full reference for the retraction notice.

**Response:** Thank you for this comment. We have removed reference number 11 that was retracted and replaced it with a more current and relevant reference.

Reference number 11 was removed and has been replaced by the following reference:

“11.	Cheng L, Hu W, Qiu B, Zhao J, Yu Y, Guan W, et al. Generation of neural progenitor cells by chemical cocktails and hypoxia. Cell Res. 2015;25: 645-646. doi: 10.1038/cr.2015.55.”

**Reviewer #2:**

It is appreciated that the authors made a strong and good-faith effort to address reviewer concerns, particularly with addition of new experiments and increased sampling sizes of original experiments. The remaining issue is the use of the word "regeneration" in a manner that is confusing. The authors state in their response that they removed the word "regeneration," yet it appears throughout the manuscript.

Other questions and concerns:

As noted previously the use of the word or concept of "regeneration" as generally understood is not supported by the data, and critically, could not be supported by the existing experimental design. The authors are asked to consider that the word "regeneration" does not appear in the body of the text of the studies that they reference as examples of "central nerve regeneration" (lines 57-61, references 9-12). The authors' data do support that their test treatment resulted in observation of "Rhodopsin-positive cell production," as correctly stated in the title of the manuscript. The authors are asked to consider using similar phrases in the body of the text rather than "regeneration." Alternatively, the authors could very precisely and thoroughly describe in the Introduction exactly what they mean by the word "regeneration" and in the Discussion describe how their data support the existence of that exactly defined outcome. This would allow the reader to understand what the authors mean when they use the word "regeneration."

**Response:** We thank the reviewer for this comment, as he/she raises an important issue. In our experimental design, SLCD treatment was performed during the process of photoreceptor cell loss; therefore, it was not possible to distinguish whether Rho expression appeared due to MC differentiation or stimulation of the remaining photoreceptor cells.

In the previous revision, we avoided the use of the word “regeneration” relating to our experimental results. However, we agree with the reviewer that the word “regeneration”, when found throughout the body of the text is misleading, as if this study leads to a conclusion that shows neural regeneration.

Thus, as per the reviewer’s suggestion, we have now removed the words “regeneration” and “regenerative” in the text and modified the manuscript as shown in the box below.

| **Page, line** | **Before** | **After** |
| --- | --- | --- |
| page 2, lines 17 | photoreceptor cell regeneration | create photoreceptor cells |
| page 3, lines 59 | In central nerve regeneration | In the central nervous system |
| page 4, lines 73-74 | photoreceptor regeneration | the differentiation from glial cells to photoreceptor cells |
| page 12, lines 294 | the regeneration of photoreceptor cells | differentiation to photoreceptor cells |
| page 14, lines 330 | photoreceptor regeneration occurred | photoreceptor cells were observed |
| page 14, lines 348-349 | the induction of cellular regenerative signals | creation of photoreceptor cells |
| page 18, lines 437 | the retinal regenerative capacity of SLCD | whether SLCD treatment produced Rho-positive cells |
| page 20, lines 491 | regeneration | differentiation |
| page 20, lines 494 | in retinal regeneration | in transformation from MCs to retinal neural cells |
| page 20, lines 497 | regeneration | production |
| page 20, lines 507 | retinal regeneration | neural differentiation in the retina |
